# Immune-related osteitis mimicking femoral head osteonecrosis during dual checkpoint blockade: a case report

**DOI:** 10.3389/fonc.2025.1621774

**Published:** 2025-10-28

**Authors:** Enrica Teresa Tanda, Agostina Lagodin D’Amato, Giovanni Rossi, Maria Maddalena Latocca, Andrea Boutros, Andrea Zanirato, Matteo Formica, Silvia Bozzano, Bruno Spina, Francesco Spagnolo

**Affiliations:** ^1^ U.O.C. Medical Oncology 2, IRCCS Ospedale Policlinico San Martino, Genoa, Italy; ^2^ Department of Internal Medicine and Medical Specialties, School of Medicine, University of Genoa, Genoa, Italy; ^3^ U.O. Clinica di Oncologia Medica, IRCCS Ospedale Policlinico San Martino, Genova, Italy; ^4^ Melanoma Institute Australia, The University of Sydney, Sydney, NSW, Australia; ^5^ Department of Integrated Surgical and Diagnostic Sciences (DISC), University of Genoa, Genoa, Italy; ^6^ Clinica Ortopedica, IRCCS Ospedale Policlinico San Martino, Genoa, Italy; ^7^ Pathology Unit, Department of Surgical Sciences and Integrated Diagnostics (DISC), University of Genoa, Genoa, Italy; ^8^ U.O.C. Anatomia Patologica Ospedaliera, IRCCS Ospedale Policlinico San Martino, Genoa, Italy

**Keywords:** osteitis, osteonecrosis, irAE, immune-related adverse event, melanoma, immune-checkpoint blockade, immunotherapy, PD-1

## Abstract

Immune checkpoint inhibitors (ICIs) have dramatically reshaped the therapeutic landscape of oncology, offering long-term survival benefits across multiple tumor types. However, ICIs are associated with a broad range of immune-related adverse events (irAEs), most of which are now well characterized and manageable. A subset of irAEs, however, remains rare, unpredictable, and poorly understood, both in terms of clinical presentation and pathogenesis. Here, we describe the case of a patient with advanced melanoma treated with combined anti-CTLA-4 and anti-PD-1 therapy who developed severe left hip pain during treatment. Imaging findings were initially suggestive of osteonecrosis of the femoral head. However, histopathological analysis of the resected femoral head revealed a dense lymphoplasmacytic infiltrate with fibrosis and vascular congestion, without evidence of bone necrosis, consistent with an immune-mediated osteitis. To our knowledge, this represents the first documented case of direct immune-related inflammation selectively affecting bone tissue during ICI therapy. Recognition of such atypical skeletal irAEs may be critical for improving diagnosis and management strategies in the expanding field of immuno-oncology.

## Introduction

The introduction of immune checkpoint inhibitors (ICIs) has revolutionized the treatment landscape of advanced melanoma (1). Recent 10-year results from the KEYNOTE-006 and CheckMate 067 trials have confirmed that approximately 50% of patients with advanced melanoma achieved long-term overall survival (OS) benefit with single agent PD-1 blockade or combined CTLA-4 and PD-1 blockade, respectively, marking an unprecedented shift in the natural history of this disease (2, 3).

Following these successes in advanced melanoma, ICIs have been rapidly integrated into the management of multiple solid and hematological malignancies, across both early and advanced disease settings.

Alongside the expanding indications for ICIs, oncologists have had to recognize and manage immune-related adverse events (irAEs), a spectrum of toxicities arising from immune hyperactivation and loss of self-tolerance.

Overall, irAEs are observed in up to 60–85% of patients receiving anti-PD-1/PD-L1 monotherapy and in over 90% of those treated with dual checkpoint inhibition (4). The most frequently involved systems include the skin (30–50%), gastrointestinal tract (15–30%), and endocrine glands (10–20%) (4).

Musculoskeletal irAEs, although less common (5–10%), typically manifest as inflammatory arthritis, polymyalgia-like syndromes, or myositis (4).

Despite the growing awareness of rheumatologic irAEs, bone tissue has historically been considered relatively spared from direct immune-mediated injury.

Only isolated cases of immune-mediated osteonecrosis of the jaw have been described, mostly in association with head and neck cancer treatments, and always histologically confirmed as true bone necrosis (5–8).

Here, we present the case of a patient with metastatic melanoma treated with combined anti-CTLA-4 and anti-PD-1 therapy, who developed a femoral head lesion initially suggestive of osteonecrosis on imaging. However, histopathological analysis revealed the absence of necrosis and the replacement of normal bone architecture by dense lymphoplasmacytic infiltration and fibrotic tissue.

To our knowledge, this represents the first reported case of an immune-mediated osteitis-like reaction involving bone during checkpoint inhibitor therapy.

## Case report

A 76-year-old woman presented to the Emergency Department in May 2023 with severe anemia. Her past medical history included hypertension, mild osteoporosis managed with calcium and vitamin D supplementation, first-degree atrioventricular block, a previous bilateral hystero-oophorectomy for benign disease, and multinodular thyroid goiter.

A contrast-enhanced CT scan revealed a 65 × 35 mm vegetating gastric lesion, along with multiple secondary pancreatic nodules located in the head (11 mm), isthmus (11 mm), and body (12 mm) of the pancreas, findings later confirmed by abdominal MRI.

Esophagogastroduodenoscopy (EGDS) identified a mammillary, ulcerated lesion in the gastric fundus. Histological analysis of biopsy samples demonstrated metastatic melanoma, BRAF wild-type (assessed by PCR and Sanger sequencing), and PD-L1 negativity (TPS <1%).

To stabilize her condition, the patient first received hemostatic radiotherapy targeting the gastric lesion to control bleeding. Subsequently, in June 2023, she initiated first-line immunotherapy with the standard combination of nivolumab (1 mg/kg) plus ipilimumab (3 mg/kg) administered every 21 days for four cycles, followed by maintenance nivolumab (480 mg) every 28 days.

The treatment was generally well tolerated, with iatrogenic hypothyroidism emerging as the sole significant irAE, effectively managed with levothyroxine replacement.

**Figure 1:**
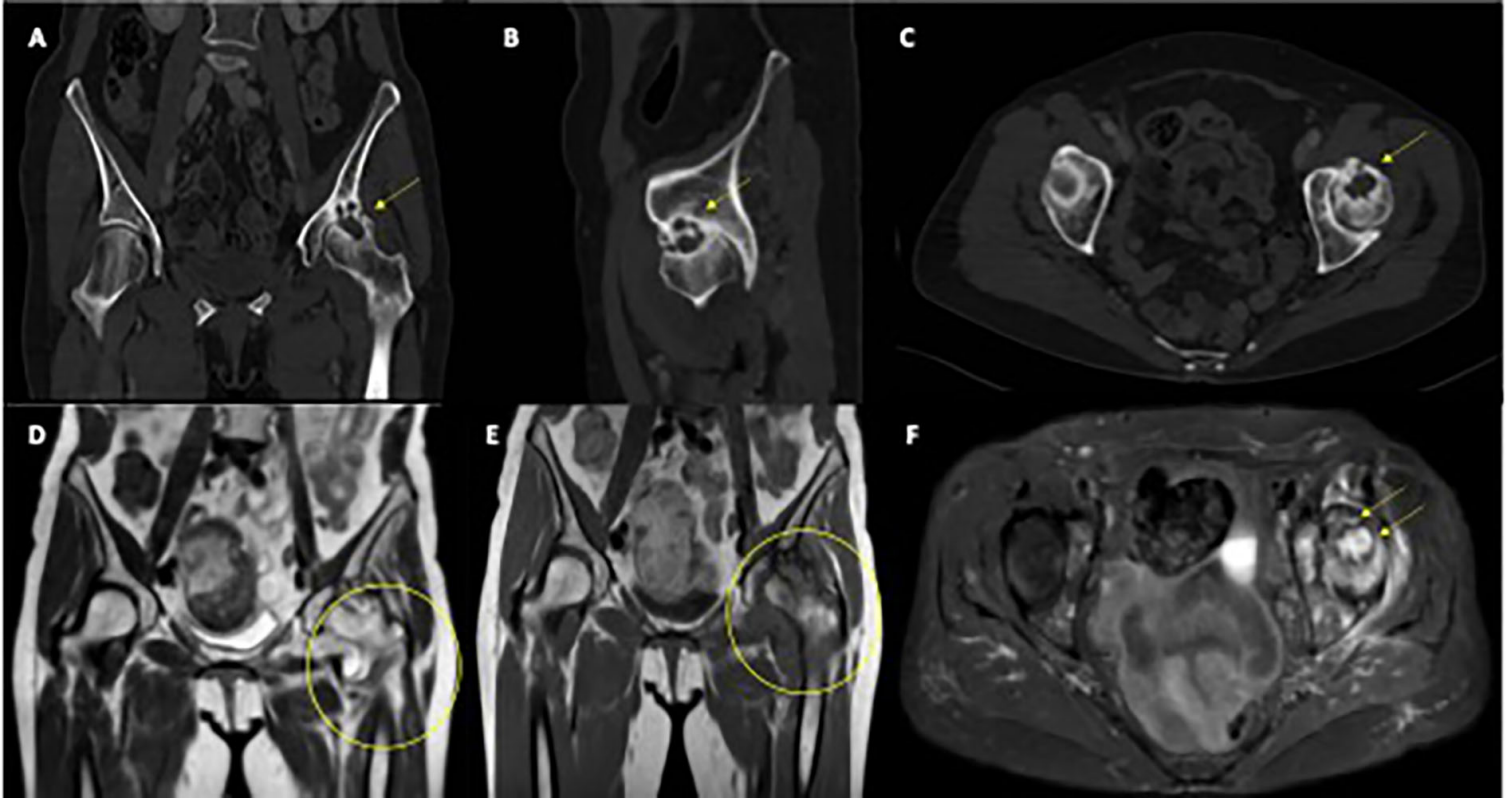
Radiological features of left femoral head osteitis during immunotherapy. **(A–C)** CT scans show progressive deformation and sclerosis of the left femoral head (yellow arrows). **(D, E)** Coronal T1-weighted MRI images demonstrate diffuse bone marrow edema and joint effusion (yellow circles). **(F)** Axial T2-weighted MRI highlights extensive marrow edema and synovial hypertrophy (yellow arrows).

First restaging scans demonstrated complete radiologic regression of both the gastric and pancreatic lesions. Incidentally, imaging also revealed severe left coxarthrosis characterized by joint space narrowing, coarse geodes, and femoral head deformation (**Figures 1A–C**).

The patient continued anti-PD-1 maintenance therapy with excellent tolerance and maintained a complete pathological response, confirmed through multiple negative EGDS-guided biopsies.

In March 2024, she developed new-onset severe left hip pain radiating to the ipsilateral lower limb.

Pelvic and spinal imaging revealed marked progression of left hip arthropathy, raising suspicion for an osteonecrotic process (**Figures 1D–F**). MRI of the left hip showed features consistent with osteonecrosis, including diffuse bone marrow edema, focal osteochondral lesions of the acetabular roof, abundant joint effusion, synovial hypertrophy, and adjacent muscle edema (**Figures 1D–F**).

An urgent orthopedic consultation led to the decision to perform left total hip replacement, which was carried out in July 2024.

Intraoperatively, a pink, soft, non-bleeding tissue was noted extending from the femoral neck to the acetabulum, associated with extensive bone erosion.

Given the patient’s history, a suspicion of metastatic melanoma prompted the surgical team to submit the femoral head for histopathological analysis.

**Figure 2:**
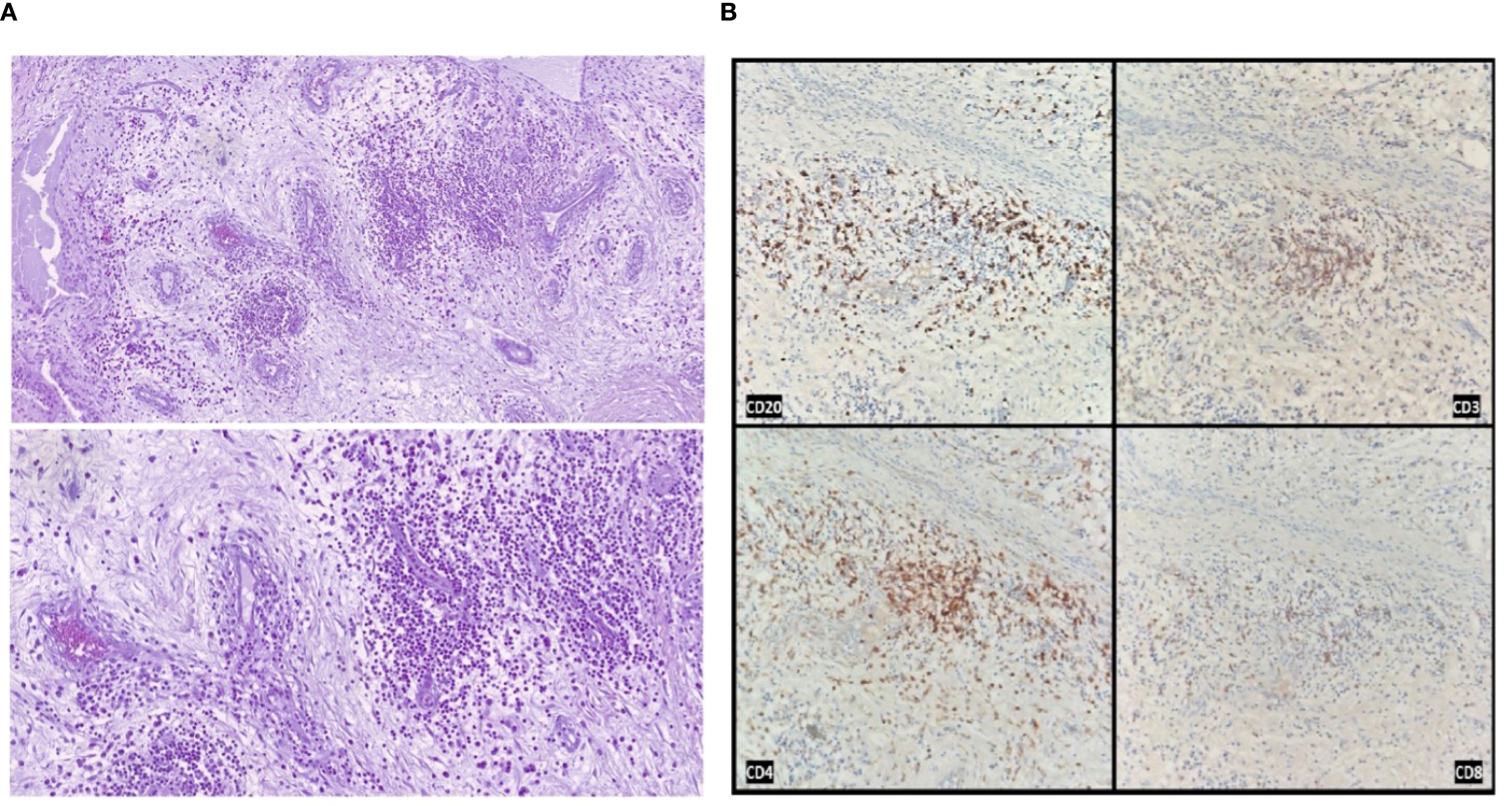
Histological and immunohistochemical analysis of the resected femoral head. **(A)** Hematoxylin and eosin staining showing areas of fibrosis with neoangiogenesis, vascular congestion, and dense lymphoplasmacytic inflammatory infiltrates, in the absence of bone necrosis (original magnification 100× and 200×). **(B)** Immunohistochemical staining demonstrating the immune infiltrate composition: CD20-positive B cells, CD3-positive T cells, with a predominance of CD4-positive over CD8-positive T lymphocytes.

Microscopic examination revealed areas of fibrosis with dilated, congested vessels and a dense lymphoplasmacytic infiltrate, without any evidence of bone necrosis (**Figures 2A, B**).

Immunohistochemical staining confirmed the absence of melanoma metastasis (SOX10 negative) and ruled out other carcinomatous lesions (p40 and AE1/AE3 cytokeratin pool negative).

Samples from the acetabulum showed fibrous tissue partially covered by synovial lining, with prominent lymphocytic follicular aggregates and marked vascular congestion.

Postoperatively, a restaging CT scan performed in July 2024 confirmed the persistence of complete radiologic response in the chest, abdomen, and brain. Following hip replacement, the patient reported complete resolution of pain and resumed normal activities. She expressed gratitude for the combined oncological and surgical care, stating she ‘can now walk again without agony’.

At her latest follow-up in January 2025, the patient remained well, reporting complete resolution of symptoms, excellent functional recovery after hip arthroplasty, and ongoing complete response of her metastatic melanoma. Anti-PD-1 therapy has not yet been resumed.

## Discussion

IrAEs are a recognized consequence of ICI therapy, stemming from immune hyperactivation and loss of self-tolerance. While a wide spectrum of organ systems can be involved, musculoskeletal irAEs are relatively uncommon and have been primarily characterized by inflammatory arthritis, myositis, or polymyalgia-like syndromes (4, 9, 10).

Direct immune-mediated injury targeting bone tissue, however, has not been previously reported.

Previous reports have described skeletal irAEs characterized by osteoporosis and resorptive bone lesions, often associated with systemic inflammatory features and inflammatory arthritis (11). Moreover, previous reports have also described and hypothesized immune-related osteonecrosis occurring during checkpoint inhibitor therapy. However, in these cases, true histological documentation was limited, and when available, findings were consistent with bone necrosis rather than active immune-mediated infiltration (5–8). Notably, no prior case reports have demonstrated dense lymphoplasmacytic infiltration replacing bone tissue in the femoral head without necrosis or tumor, as observed in our patient.

In this case, a patient with metastatic melanoma treated with combined CTLA-4 and PD-1 blockade developed progressive left hip pain, with imaging findings suggestive of osteonecrosis of the femoral head. Histopathological examination of the surgically resected femoral head, however, revealed a completely different pattern: the absence of bone necrosis, replaced by dense lymphoplasmacytic infiltration, fibrosis, and vascular congestion, without evidence of bone cell death or trabecular collapse.

This histological profile was highly suggestive of an active immune-mediated inflammatory process, an “osteitis “, rather than classical ischemic avascular necrosis (AVN).

Moreover, the patient had no known risk factors typically associated with AVN, such as chronic corticosteroid use, alcohol abuse, trauma, or hematologic conditions (12). The combination of clinical, radiological, and histopathological findings strongly supports the hypothesis of an immune-mediated mechanism triggered by checkpoint inhibition.

Beyond clinical presentation, important histopathological and pathophysiological differences distinguish AVN from the immune-mediated osteitis observed in our patient.

Classical AVN is characterized by ischemic bone infarction, leading to trabecular collapse, empty osteocytic lacunae, and eventual joint destruction, typically without significant inflammatory infiltration. In contrast, immune-mediated osteitis presents with preserved bone structure, dense lymphoplasmacytic infiltrates, neoangiogenesis, and active chronic inflammation without evidence of bone necrosis. These fundamental differences, summarized in **Table 1**, support the interpretation of this event as a novel skeletal irAE distinct from both classical AVN and medication-related osteonecrosis of the jaw (ONJ). In selected cases, ^99m^Tc-methylene diphosphonate (MDP) bone scintigraphy may provide additional diagnostic information. In fact, early AVN often presents as a photopenic (“cold”) lesion due to impaired blood supply, whereas immune-mediated osteitis is expected to show increased radiotracer uptake reflecting enhanced bone turnover. Although not performed in our patient, this modality could be considered when MRI findings are inconclusive, and its absence represents a limitation of our report.

**Table 1:** Comparison between classical osteonecrosis and immune-mediated osteitis.

Feature	Classical osteonecrosis (12)	Immune-mediated osteitis (this case)
Typical location	Femoral head, knee, proximal humerus*	Unknown
Pathogenesis	Ischemia due to vascular impairment (trauma, corticosteroids, alcohol)	Aberrant immune activation triggered by immune checkpoint inhibitors
Predisposing factors	Corticosteroids, alcohol, trauma, hematologic diseases	Immunotherapy with ICIs
Radiological features	Subchondral collapse, sclerosis, geodes, joint space narrowing	Bone marrow edema, joint effusion, synovial hypertrophy, no subchondral collapse
Histopathology	Empty osteocytic lacunae, trabecular collapse, absence of viable osteocytes	Dense lymphoplasmacytic infiltrates, fibrosis, preserved bone structure, neoangiogenesis
Inflammatory pattern	Minimal or absent	Active chronic inflammation with follicular lymphoid aggregates
Presence of neoplastic cells	Not applicable (unless metastasis present)	None (SOX10-negative, p40-negative, AE1/AE3-negative)
Clinical course	Progressive joint deterioration, high risk of collapse; surgical replacement often required	Symptom improvement after surgery; maintained oncological remission

AVN, avascular necrosis; ICIs, immune-checkpoint inhibitors; SOX10, SRY-box transcription factor 10; p40, ΔNp63 isoform; AE1/AE3, cytokeratin markers pool.

*Classical osteonecrosis (AVN) refers to ischemic necrosis of load-bearing bones (such as the femoral head, knee, and humerus) due to vascular impairment. It does not include medication-related osteonecrosis of the jaw (ONJ), which has a distinct pathogenesis primarily involving antiresorptive therapies and local infection.

One alternative hypothesis could be that of a solitary melanoma bone metastasis undergoing complete pathological response following immunotherapy. However, several elements argue against this possibility.

In fact, baseline staging scans revealed no evidence of skeletal involvement, and no other bone lesions were detected throughout follow-up. Histopathological analysis showed no residual neoplastic cells, confirmed by negative staining for melanoma markers (SOX10) and epithelial markers (p40, AE1/AE3).

Furthermore, in cases of complete pathological response of melanoma metastases, the expected histological findings would typically include fibrotic tissue and occasional melanosis - remnants of regressed tumor - but not an organized, dense, lymphoplasmacytic inflammatory infiltrate (13).

The presence of active, structured chronic inflammation, with follicular aggregates and vascular congestion, is much more consistent with an ongoing immunologic process than with scar tissue from tumor regression. Nonetheless, the possibility of an isolated melanoma metastasis undergoing complete immune-mediated regression cannot be completely ruled out. Given the broad spectrum of femoral head pathologies, we acknowledge that this inflammatory process could be unrelated to ICI therapy. However, the strong temporal association, absence of conventional risk factors, and distinctive histopathological features make an immune-mediated mechanism the most plausible explanation in this case.

This case highlights a previously unrecognized manifestation of checkpoint inhibitor toxicity, suggesting that bone tissue can be a direct target of immune-mediated inflammation (14). Recognition of this phenomenon is crucial because it may mimic more common conditions such as osteonecrosis but has a fundamentally different pathogenesis and implications for management.

To our knowledge, this is the first reported case of immune-mediated osteitis during ICI therapy. Although a causal link cannot be definitively proven and an unrelated etiology cannot be excluded, the temporal association, absence of conventional risk factors, and distinctive histopathological findings make an immune-mediated mechanism the most plausible explanation. Given the shared mechanism of action of ICIs, a class effect cannot be ruled out. **Table 2** summarizes key features, differential diagnoses, and practical management considerations. Further research is needed to clarify its incidence, pathogenesis, and optimal management.

**Table 2:** Key clinical features and management considerations for suspected immune-mediated osteitis during immune checkpoint inhibitor therapy (orthopedic perspective).

Step	Orthopedic perspective	Practical notes for oncologists
When to suspect	New focal bone/joint pain (esp. load-bearing joints) during ICI; no trauma history; unusual MRI pattern (bone marrow edema without subchondral collapse)	Consider MRI early if pain persists >2–3 weeks
Initial workup	Focused history (trauma, alcohol, corticosteroids), exam for joint effusion; screen infection/inflammation	Basic labs (CBC, CRP/ESR) to exclude infection; plain X-ray as first line where appropriate
Differential diagnosis	Avascular necrosis, metastasis, septic arthritis, inflammatory arthritis, metabolic bone disease	Maintain broad ddx early; involve ortho/rheum/radiology as needed
Key imaging clues	Preserved bone structure, marrow edema, synovial hypertrophy, absence of necrotic bone on MRI	Coordinate radiology-orthopedic review. Ask radiology to comment explicitly on features arguing against AVN and metastasis
Histopathology clues	Dense lymphoplasmacytic infiltrate, fibrosis, vascular congestion, preserved trabeculae	Biopsy/surgical specimen only when infection or metastasis cannot be ruled out or surgery is indicated
Management principles	Symptom control; joint-preserving options vs arthroplasty depending on structural damage; avoid empiric antibiotics if infection unlikely	Multidisciplinary review (oncology, orthopedics, radiology, pathology ± rheumatology)
ICI management	Orthopedic input on structural risk; defer ICI decisions to oncology	Case-by-case decision on ICI hold/resume based on cancer status and severity of bone involvement, but mainly cease ICI

ICI, immune checkpoint inhibitor; AVN, avascular necrosis; CBC, complete blood count; CRP, C-reactive protein; ESR, erythrocyte sedimentation rate; MRI, magnetic resonance imaging.

## Conclusions

In conclusion, we describe what appears to be the first reported case of an immune-mediated osteitis involving the femoral head, likely induced by combined anti-CTLA-4 and anti-PD-1 therapy.

This adverse event may be significantly underdiagnosed or underreported, given that hip arthroplasty is a common procedure among elderly patients, including those with a history of cancer, and femoral heads are not routinely subjected to detailed histopathological analysis.

Recognition of such atypical immune-related events is crucial for improving our understanding of the full spectrum of skeletal toxicities associated with immune checkpoint inhibitors.

Further studies are warranted to elucidate the pathogenesis of this phenomenon and to define optimal strategies for diagnosis and management.

## Data Availability

The raw data supporting the conclusions of this article will be made available by the authors, without undue reservation.
